# Chemical Communication between Giant Vesicles and Gated Nanoparticles for Strip-Based Sensing

**DOI:** 10.1021/acs.nanolett.4c04022

**Published:** 2024-10-23

**Authors:** Jordi Ventura-Cobos, Estela Climent, Ramón Martínez-Máñez, Antoni Llopis-Lorente

**Affiliations:** † Instituto Interuniversitario de Investigación de Reconocimiento Molecular y Desarrollo Tecnológico (IDM), Universitat Politècnica de València, Universitat de València, Camino de Vera s/n, 46022 València, Spain; ‡ CIBER de Bioingeniería, Biomateriales y Nanomedicina (CIBER-BBN), Instituto de Salud Carlos III, 28029 Madrid, Spain; § Unidad Mixta de Investigación en Nanomedicina y Sensores, Universitat Politècnica de València, Instituto de Investigación Sanitaria La Fe (IISLAFE), Avenida Fernando Abril Martorell 106, 46026 Valencia, Spain; ∥ Unidad Mixta UPV-CIPF de Investigación en Mecanismos de Enfermedades y Nanomedicina, Universitat Politècnica de València, Centro de Investigación Príncipe Felipe, C/Eduardo Primo Yúfera 3, 46100 Valencia, Spain; ⊥ Departamento de Química, Universitat Politècnica de València, Camino de Vera s/n, 46022 Valencia, Spain

**Keywords:** Chemical Communication, Nanoparticles, Giant Vesicles, Sensing, Toxin

## Abstract

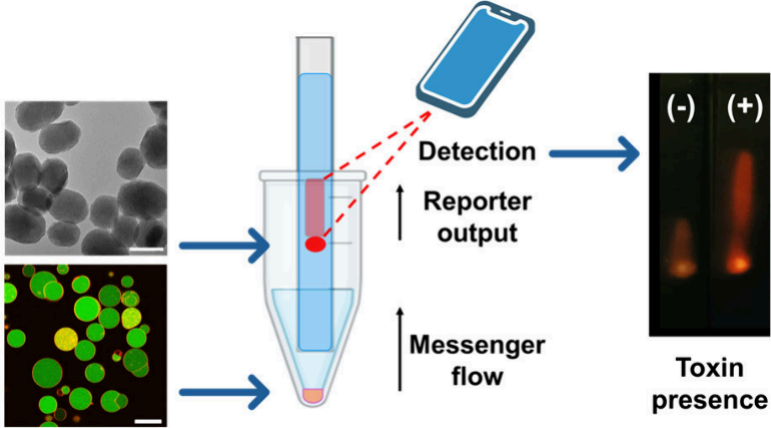

Inspired by nature, the development of artificial micro/nanosystems capable of communicating has become an emergent topic in nanotechnology, synthetic biology, and related areas. However, the demonstration of actual applications still has to come. Here, we demonstrate how chemical communication between micro- and nanoparticles can be used for the design of sensing systems. To realize this, we synergistically combine two different types of particles: i.e., giant unilamellar vesicles (GUVs) as senders and gated mesoporous nanoparticles as receivers. The use of engineered GUVs allows the detection of analytes based on responsive membranes, while the use of gated nanoparticles allows a straightforward application on test strips with smartphone-based detection. In addition, we demonstrate that the combined communication system exhibits signal amplification and its application in real samples employing the bacterial toxin α-hemolysin as target analyte. Altogether, our report presents a new route for engineering sensing systems based on the combination of communicative micro/nanoparticles.

The engineering of nanoscale particles, nanostructured materials and cell mimics able to exhibit chemical communication with other entities via the exchange of molecular messengers is a key challenge in nanotechnology, synthetic biology and related areas.^1−5^ Pioneering studies have demonstrated communication between proteinosome or coacervate microparticles via the programmed exchange of DNA strands.^6−11^ In addition, communication between enzyme-loaded lipid-based artificial cells was achieved via production and secretion of enzymatic substrates.^12−14^ A more complex approach relies on the encapsulation of protein synthesis machinery in lipid vesicles which activates the release of entrapped messengers in response to external stimuli.^15−18^ In the area of nanotechnology, special interest has been placed on the development of gated nanosystems — i.e., mesoporous scaffolds functionalized with stimuli-responsive ensembles (gatekeepers) that control the release of cargo. Different models of communication between gated nanoparticles have been demonstrated in which the cargo released by one set of nanoparticles activates the gatekeepers of the next set of nanoparticles in a programmed pathway.^19−23^ A recent direction leverages engineered nanoparticles or artificial cells to establish communication with living cells.^24−26^ Despite progress, the communication examples reported so far are proof-of-concept demonstrations or fundamental investigations. In particular, the potential of engineering communication between micro/nanosystems for sensing applications is unexplored.

In a different context, the design of stimuli-responsive lipid vesicles (liposomes) has received significant attention because of their potential for controlled drug delivery. A variety of liposome formulations responsive to pH, enzymes, small molecules, or external stimuli (light, temperature) have been designed, based on either the permeation or degradation of the phospholipid membrane.^27−29^ Similarly, responsive polymersomes (polymeric vesicles) have also been developed.^30−32^ Whereas nanoliposomes are typically used for nanomedicine applications, giant unilamellar vesicles (GUVs, 1–100 μm) are recognized as a versatile synthetic platform with size and bilayer structure reminiscent to those of natural cells.^33^ Interestingly, GUVs allow the encapsulation of large amounts of diverse molecular and macromolecular cargoes, such as stimuli-responsive protein synthesis machinery.^34−36^

Inspired by the role of chemical communication in nature, here we demonstrate that engineered communication between micro/nanoparticles can be used for the design of sensing systems in which senders emit a chemical messenger to activate the release of a reporter from distant receivers (Scheme 1A). As far as we know, this strategy has not been reported before in the areas of sensing and chemical communication. Two different types of particles—i.e., GUVs as senders and gated nanoparticles as receivers—are synergistically combined in our sensing system. The use of engineered GUVs allows us to leverage analyte-membrane interactions to translate the presence of target analyte molecules into the emission of a chemical messenger. In turn, the use of engineered gated nanoparticles, able to respond to the chemical messenger released by GUVs, allows the translation of the presence of the analyte into the release of a colorimetric/fluorometric reporter ([Ru(bpy)_3_]Cl_2_) and the application of the communication system on test-strips (Scheme 1B). In our setup, test strips functionalized with gated nanoparticles are immersed in a test tube containing GUVs and the target sample. The chemical messenger sent by GUVs flows through the strip to the nanoparticles’ spot, and subsequently the released reporter migrates by capillarity. Using a smartphone coupled with a 3D-printed case containing proper LEDs and optical filters (Figure S1), the signal produced by the reporter is correlated with the presence of analyte in the sample. We showcase the feasibility of this approach using the toxin α-hemolysin as the target analyte. In fact, α-hemolysin in milk samples has been proposed as a biomarker of bovine mastitis, a disease that causes loses up to $31.4 billion per year.^37,38^ In particular, to achieve communication, GUVs are loaded with acetythiocholine (ATCh) as a chemical messenger and formulated to enable the insertion of α-hemolysin in their membrane. In turn, gated nanoparticles are based on mesoporous silica loaded with a dye in their pores and capped with the enzyme acetylcholinesterase (AChE) via pH sensitive linkages. Our results demonstrate the potential of engineering chemical communication between micro/nanosystems responsive to target (bio)molecules and open new venues in the area of sensing.

**Scheme 1:**
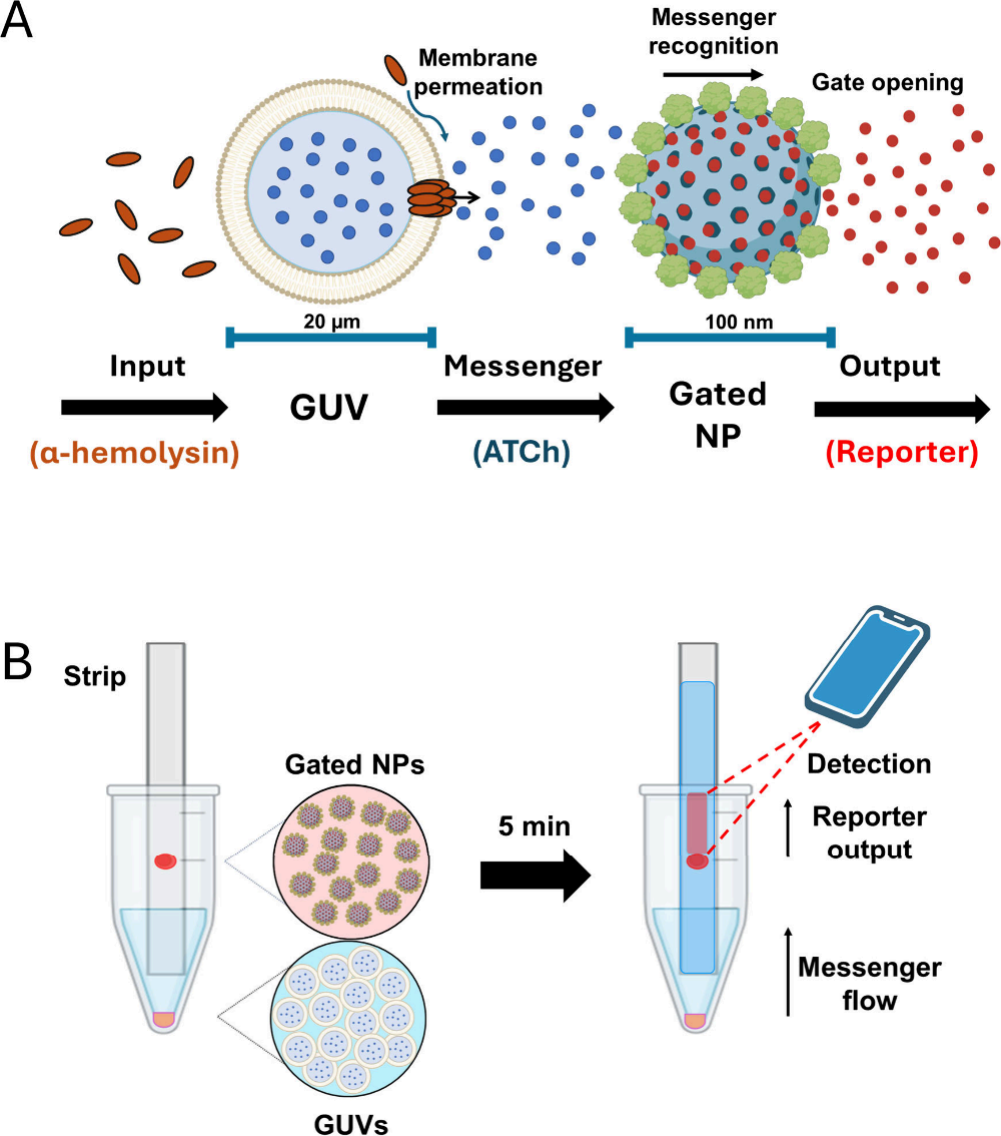
Schematic representation of chemical communication between cell-sized vesicles (GUVs) and gated nanoparticles (NPs). A) In response to an input, membrane permeation enables the release of an entrapped chemical messenger (ATCh) which is recognized by the gated nanoparticles. The subsequent opening of the molecular gate leads to the release of reporter molecules as the output of the communication. B) The communication paradigm is implemented on test strips, as depicted. GUVs interact with the target sample in a test tube, and messenger flows through the strip to the nanoparticles. The reporter output is detected using a smartphone-based setup.

In the first step, we set out to prepare GUVs with the ability to encapsulate and release their cargo in response to α-hemolysin. In order to assemble GUVs, the droplet transfer method was used (see SI for details, Figure S2).^39^ The lipid composition of GUV’s membrane is an important factor that influences the physiochemical properties of vesicles.^40,41^ GUVs of *ca.* 20 μm were prepared from a mixture of phospholipids (DOPC/POPC) at equimolar concentration based on our previous studies.^42,43^ The optimal proportion of cholesterol for α-hemolysin triggered cargo release was studied by encapsulating the fluorophore HPTS in various populations of GUVs with a molar ratio of 0%, 30% and 50% of cholesterol in their membranes (total lipid concentration was fixed at 20 μmol). When we attempted to prepare GUVs with 70% cholesterol, we noticed that the yield was drastically reduced (Figure S3). Interestingly, the size and yield of GUVs were not affected when the cholesterol molar ratio was adjusted from 0 to 50%. To evaluate the response to α-hemolysin, an aliquot of each GUV preparation was transferred to a microscope chamber and visualized before and after 10 min incubation with α-hemolysin (20 μg mL^–1^, 0.6 μM). As shown in confocal images (Figure 1), GUVs with the highest proportion of cholesterol (50%) showed the best response to α-hemolysin, with 92% of the population (*N* > 250) releasing their cargo—in turn, GUVs with 0% and 30% cholesterol showed cargo release in 34% and 75% of the vesicle population, respectively (*N* > 250). The percentage of GUVs releasing their cargo increased after 60 min of incubation with α-hemolysin (Figure S4). Subsequently, GUVs with 50% cholesterol on their membrane and 80 mM ATCh (chemical messenger) incorporated in their inner phase were prepared. These GUV samples were then incubated (10 min) with an aqueous solution containing (or not, as a control) α-hemolysin (100 μg mL^–1^, 3 μM), followed by quantification of ATCh in the supernatant. A remarkable release of ATCh (0.9 ± 0.1 mM) was determined (based on Ellman’s method)^44^ after α-hemolysin triggered cargo release, while minimal leakage was detected in control samples (see SI for details, Figure S5). These results indicated that our formulated GUVs allow proper encapsulation of ATCh and the fast release of this chemical messenger in response to α-hemolysin in their environment.

**Figure 1:**
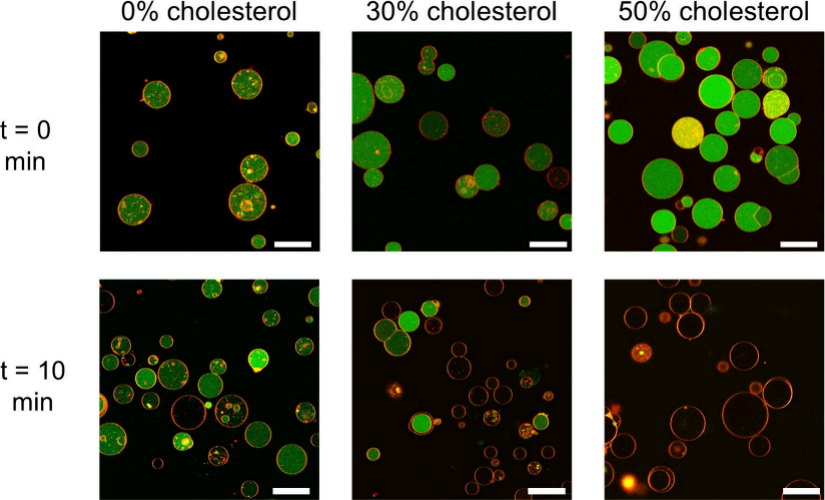
Characterization of responsive GUVs capable of releasing a chemical messenger from their lumen in response to the presence of α-hemolysin. Confocal images of (rhodamine) membrane-labeled GUVs formulated with different molar ratios of cholesterol, loaded with HPTS (green, 50 μM) before and after 10 min incubation with α-hemolysin (20 μg mL^–1^, 0.6 μM). With 50% cholesterol, the entrapped cargo (HPTS) is released, which is indicative of the proper insertion of pore-forming α-hemolysin. Scale bars represent 25 μm.

In our design, mesoporous nanoparticles are functionalized with AChE molecules, which act as both pore capping agents (preventing cargo release) and effector control units able to process the presence of ATCh in the environment and trigger reporter release (Figure 2A). In this molecular information transduction mechanism, AChE transforms the chemical messenger ATCh (input) into thiocholine and acetic acid (internal signal), producing a local pH reduction that opens the molecular gate. First, mesoporous silica nanoparticles were synthesized by the sol–gel template method and characterized using different techniques (see SI for details and Figures S6–S10). Transmission electron microscopy (TEM) imaging confirmed the formation of nanoparticles with an average diameter of 104 ± 21 nm (*N* = 80), porous structure, and spheroidal morphology (Figure 2B). Nanoparticles were subsequently treated with (3-glycidyloxypropyl) trimethoxysilane and 3-aminophenyl boronic acid to functionalize their surface with phenyl boronic acid moieties. Pores were loaded with a ruthenium complex ([Ru(bpy)_3_]Cl_2_) as a chromo-fluorogenic reporter molecule (which provides constant absorbance and fluorescence intensity regardless of the pH (Figure S11)). Finally, nanoparticles were capped with the enzyme AChE through pH-sensitive boronic ester linkages.^45^ The surface modification was confirmed by zeta potential measurements, with an increase (from −42.7 to −24.6 mV) after functionalization with the phenylboronic acid moieties and a decrease to −38.7 mV after the attachment of AChE. The total amount of encapsulated dye was estimated to be 76.5 μg mg^–1^ by UV–vis spectroscopy after exposing the nanoparticles to acidic conditions. In addition, the fluorescence intensity (λ_exc_ = 453 nm, λ_em_ = 590 nm) recovered from the nanoparticles due to [Ru(bpy)_3_]Cl_2_ delivery increased when reducing the pH from 7.5 to 5 (Figure S12).

**Figure 2:**
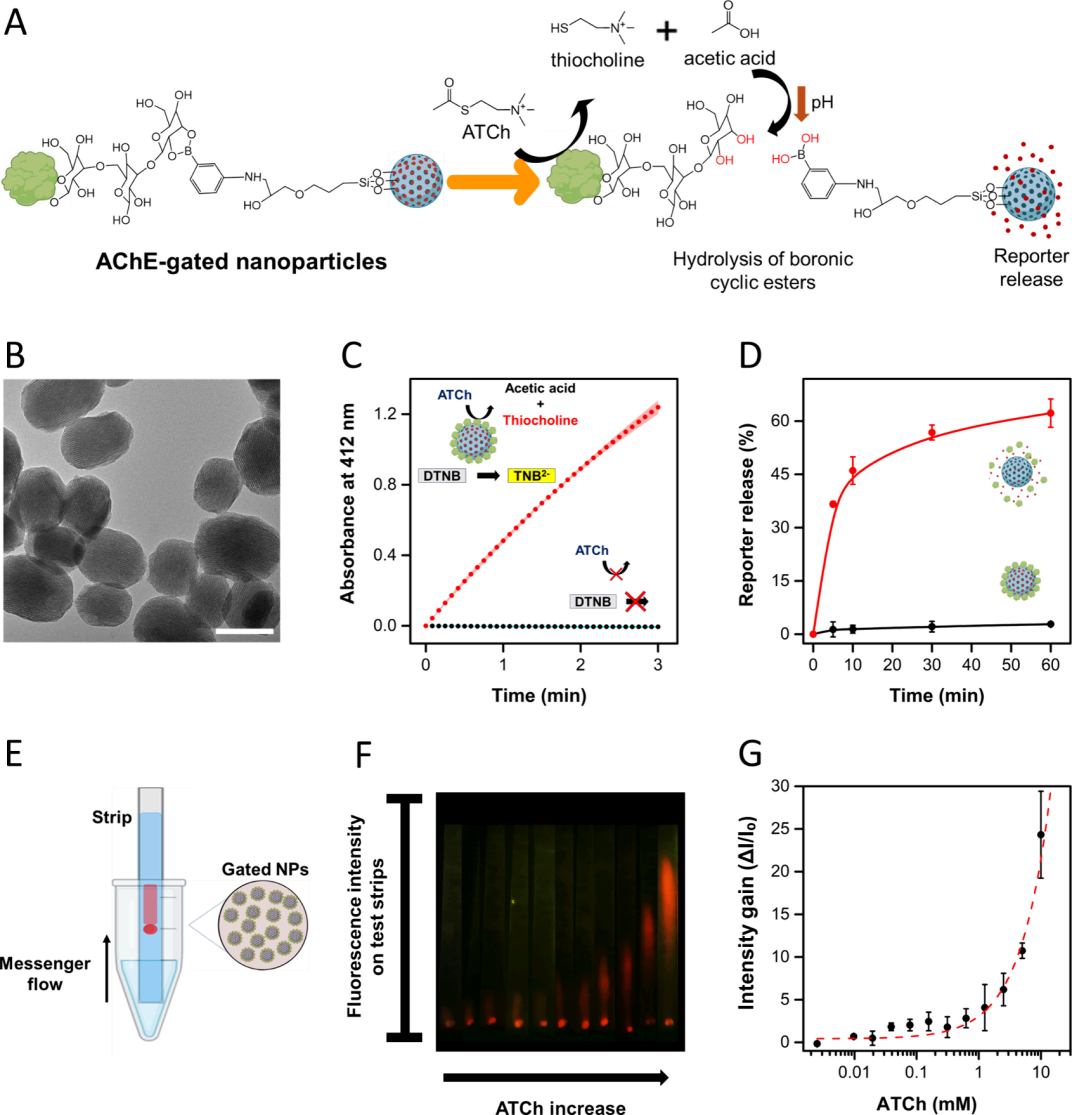
Schematic and characterization of gated nanoparticles responsive to the chemical messenger ATCh. A) Schematic of the cargo release mechanism of AChE-gated nanoparticles, triggered by the presence of ATCh. B) TEM image of mesoporous silica nanoparticles. Scale bar represents 100 nm. C) Increase in absorbance at 412 nm (corresponding to TNB^2–^ formation) due to AChE activity as a function of time in the absence (black, control) and in the presence of gated nanoparticles (red, 12.5 μg mL^–1^). DTNB is used as a substrate that reacts with thiol-containing molecules such as thiocholine produced from the transformation of ATCh by the action of AChE-functionalized nanoparticles. D) Kinetics of reporter release from gated nanoparticles in aqueous solution in the absence (black) and in the presence of chemical messenger (20 mM ATCh, red). E) Schematic of the lateral flow assay with gated nanoparticles deposited on strips. F) Collage of the photographs registered with a smartphone, showing reporter release from nanoparticles on strips upon incubation (5 min) with increasing concentrations of chemical messenger (ATCh) (from 0 to 10 mM). G) Quantified intensity gain (corresponding to reporter intensity on the elution area) as a function of ATCh concentration. The intensity gain was calculated as the ratio Δ*I*/*I*_o_, where Δ*I* corresponds to the difference between sample intensity and blank intensity (*I*_s_–*I*_o_), and *I*_o_ corresponds to the intensity of the blank (0 mM concentration, first point in Figure 2F). In all cases, error bars correspond to the s.d. of three independent experiments.

The ability of the nanoparticles to respond to ATCh was evaluated using several methods. First, the ability to transform ATCh of our AChE-gated nanoparticles was monitored by following the transformation of DTNB (a chemical that reacts with thiol-containing molecules such as thiocholine) to TNB^2–^ over time (Figure 2C). A rapid formation of TNB^2–^ was observed only when AChE-gated nanoparticles were present. From the slope of the absorbance increase, an AChE activity of 2464 ± 62 enzymatic units (U) per g of nanoparticles was determined. This demonstrated the attachment and functionality of the enzyme on the surface of the nanoparticles. The amount of attached AChE was estimated to be 13.7 μg per mg of nanoparticles, by comparison with the determined activity of commercial AChE. Second, the ability of AChE-gated nanoparticles to release its cargo upon sensing of ATCh was monitored by following the absorbance of the reporter dye in the supernatant in the presence and absence of ATCh (20 mM) for 1 h (Figure 2D). A significant payload release was registered in the presence of ATCh after just 5 min, and after this, it increased slowly with time, reaching 62% of the total loaded dye after 60 min. In contrast, the nanoparticles remained closed, and negligible output was registered in the absence of ATCh.

In a further step, we wanted to integrate our AChE-gated nanoparticles on the lateral flow test strips. Strip-based lateral flow assay tests employ liquid samples that flow by capillarity through a porous strip, similar to the successful tests developed during the COVID-19 pandemic.^46,47^ The strip typically contains (bio)molecules (often antibodies) that produce an optical output in the presence of the analyte. Notwithstanding, it is of high interest in this field to develop new strip-based strategies that go beyond the use of antibodies. For these experiments, we selected glass microfiber membranes (GF/C grade) that allow for the transport of small molecules through capillary forces. This material allows for the fast flow of the solvent and ensures a low background signal. Strips were prepared by cutting individual pieces with a size of 0.5 × 5 cm with 5 μg of the gated nanoparticles deposited at 1 cm from the border. The nanoparticles were deposited on test strips by manually placing a nanoparticle suspension (5 μL, 1 mg mL^–1^) using a pipet. Afterward, the strips were dried at room temperature for 5 min, which allows the physical adsorption of the nanoparticles as previously reported.^48^ The glass microfiber filaments of strips prevent the movement of the nanoparticles while allowing the flow of water and reporter molecules. To study if AChE-gated nanoparticles would maintain their functionality, strips were immersed in a solution containing different concentrations of chemical messenger (ATCh) as represented in Figure 2E. After 5 min of elution, photographs of the strips (Figure 2F) were taken with a smartphone connected to a homemade 3D-printed case containing a 465 nm LED as excitation source (see SI for details, Figure S1). Using the software ImageJ, the reporter intensity on the elution area was extracted and plotted as intensity gain ((sample intensity-blank intensity)/blank intensity). As observed in the photographs and the corresponding quantification (Figure 2G), the reporter signal and intensity gain progressively increased in the 0.2 to 10 mM ATCh concentration range. The LOD was calculated to be 0.18 mM ATCh, which makes the amount of ATCh released from a single GUV tube preparation (0.9 ± 0.1 mM ATCh) suitable for strip-based detection of α-hemolysin (*vide infra*).

After studying GUVs and gated nanoparticles individually, we wanted to test the chemical communication network between both types of particles. To do so, different preparations of ATCh-loaded GUVs were incubated for 10 min in the presence and absence of α-hemolysin (100 μg mL^–1^, 3 μM). Then, the supernatant (containing the released content from GUVs) was transferred to a new tube containing gated nanoparticles, since nanoparticle’s release is measured under harsh conditions (strong centrifuge and stirring) that might break GUVs. As shown in Figure 3A, a significant difference in reporter release between presence and absence of α-hemolysin was registered after just 5 min. In order to confirm that reporter release happens as a result of communication and to discard unintended crosstalk, we performed additional control experiments with GUVs without chemical messenger (ATCh). When using these messenger-lacking GUVs, the addition (or not) of α-hemolysin did not result in significant differences in reporter release from the gated nanoparticles (Figure 3B). Thus, reporter release from gated nanoparticles was produced only when the communication system was complete, i.e. in the presence of both the input (α-hemolysin) to start the communication and GUVs loaded with chemical messenger (ATCh); if any of these components is lacking, communication is disrupted.

**Figure 3:**
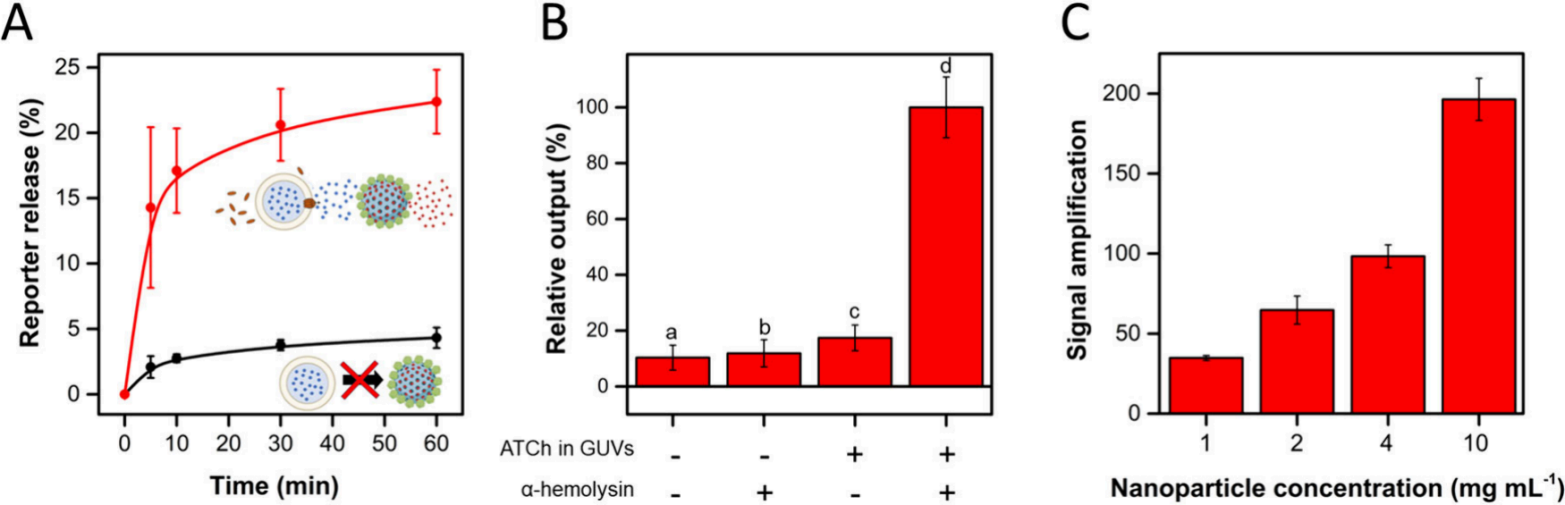
Chemical communication between GUVs and gated nanoparticles in aqueous media. A) Kinetics of reporter release from gated nanoparticles in aqueous solution, when a suspension of GUVs was treated (red line) or not (black line) with α-hemolysin (100 μg mL^–1^, 3 μM) for 10 min. Reporter absorbance was measured at λ = 452 nm. B) Relative reporter output from gated nanoparticles in communication experiments (60 min incubation) when lacking diverse components as indicated: (a) absence of both chemical messenger in GUVs and α-hemolysin input, (b) absence of chemical messenger in GUVs, (c) absence of α-hemolysin input, (d) complete system with chemical messenger (ATCh)-loaded GUVs and presence of α-hemolysin input. C) Signal amplification of the system, represented as the molar ratio between the reporter output and input (α-hemolysin), for different concentrations of gated nanoparticles after 60 min incubation. In all cases, error bars correspond to the s.d. of three independent experiments.

In the context of developing chemical communication between micro- and nanosystems for sensing purposes, it is of interest to develop strategies to maximize the output of reporter molecules. In this regard, we decided to study the effect of nanoparticle concentration on the registered signal. To quantify this, we determined signal amplification as the molar ratio between reporter output and α-hemolysin input in communication experiments in which we varied the quantity of nanoparticles in the system. The amount of GUVs (one batch) and α-hemolysin input (100 μg mL^–1^, 3 μM), and therefore the amount of available chemical messenger (released from GUVs), were kept fixed in all cases. The number of released reporter molecules was calculated from spectrophotometry measurements by applying the Beer–Lambert law. As depicted in Figure 3C, a large signal amplification was registered in all cases in the 1 to 10 mg·mL^–1^ nanoparticle concentration range. Interestingly, the determined signal amplification increased from 35 to 196 when increasing the nanoparticle concentration. This correlation between nanoparticle concentration and signal amplification can be ascribed to the chemical messenger, leading to the activation of a higher number of nanoparticles when more nanoparticles are available.

Motivated by the results observed in solution, we set out to implement this chemical communication paradigm in a strip-based lateral flow assay. In our assay, GUVs were first placed in a test tube and incubated with α-hemolysin-containing samples for 10 min. We run different experiments by exposing GUVs to concentrations of α-hemolysin that ranged from 12.5 μg mL^–1^ (0.37 μM) to 100 μg mL^–1^ (3 μM). Then, test strips containing 5 μg of deposited nanoparticles (prepared as described above) were immersed in the solution, as represented in Figure 4A. Interestingly, GUVs naturally sink at the bottom of the tube during the 10 min of incubation due to the higher density of their inner phase, preventing the absorption and disruption of GUVs by the borosilicate glass strips. The solution was let to flow for 5 min and then photographs were taken with our smartphone-based setup. As observed in the photographs (Figure 4B) and the corresponding quantification (Figure 4C), the reporter output on the strip increased with increasing α-hemolysin concentration. By fitting a linear trend line over the tested concentration range, a limit of detection of 15.1 μg mL^–1^ (0.45 μM) was calculated, which is below the reported α-hemolysin concentrations in mastitic milk.^37,49^ Altogether, these results demonstrated that the communication system between GUVs and gated nanoparticles for sensing can be implemented in test strips.

**Figure 4:**
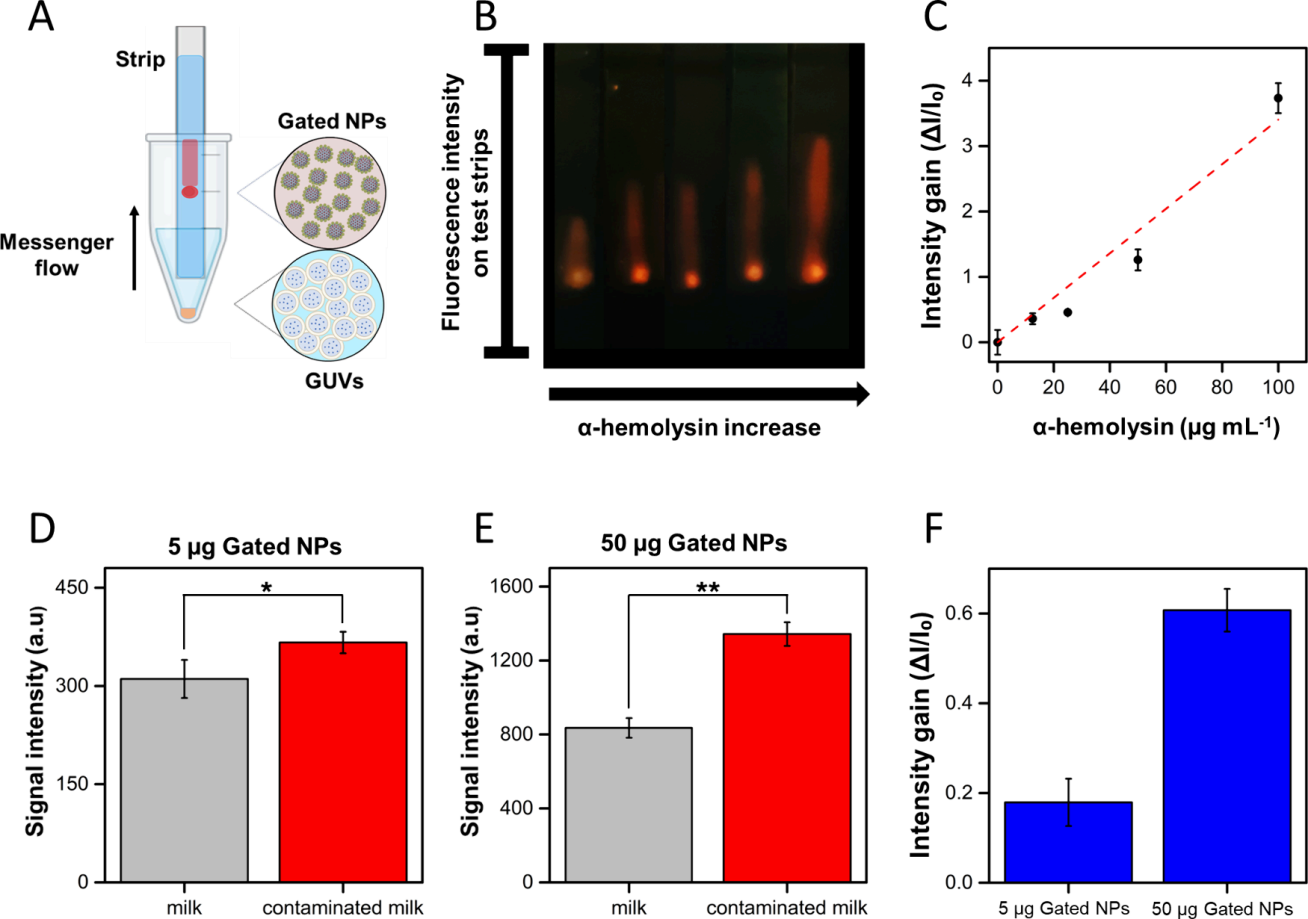
Chemical communication between GUVs and gated nanoparticles on test strips. A) Schematic of the lateral flow assay, where GUVs interact with the target sample in a test tube, and the messenger flows through the strip to the gated nanoparticles triggering the release of the reporter. B) Collage of the photographs registered with a smartphone, showing reporter release from nanoparticles on strips upon communication with GUVs exposed to increasing concentrations of α-hemolysin. From the leftmost strip (0 μg mL^–1^ α-hemolysin) to the strip on the far right (100 μg mL^–1^ α-hemolysin). C) Quantified intensity gain (corresponding to reporter intensity on the elution area) as a function of α-hemolysin concentration. The intensity gain was calculated as the ratio Δ*I*/*I*_o_, where Δ*I* corresponds to the difference between sample intensity and blank intensity (*I*_s_–*I*_o_), and *I*_o_ corresponds to the intensity of the blank (without α-hemolysin, first point in Figure 4B). D-E) Quantified reporter intensity when using samples of natural milk and α-hemolysin-contaminated milk as inputs for the communication system between GUVs and different quantities of gated nanoparticles (5 and 50 μg, respectively) on strips (*p < 0.05, **p < 0.01). α-Hemolysin concentration in contaminated milk was 100 μg mL^–1^ (3 μM). F) Quantified intensity gain (corresponding to reporter intensity on the elution area) when employing 5 and 50 μg of gated nanoparticles. The intensity gain was calculated as the ratio Δ*I*/*I*_o_, where Δ*I* corresponds to the difference between the sample containing contaminated milk and the sample containing non-contaminated milk (*I*_s_–*I*_o_), and *I*_o_ corresponds to the intensity of the sample containing non-contaminated milk. In all cases, error bars correspond to the s.d. of three independent experiments.

Inspired by the performance of the system on test strips, we aimed at employing our GUV/gated nanoparticle-communication-based assay for the detection of α-hemolysin in milk samples. As we could not obtain access to bovine mastitis samples, we employed whole cow milk spiked with α-hemolysin. In a first attempt, we followed the same procedure as previously described, using 5 μg of gated nanoparticles deposited on each strip. Experiments were run in parallel with natural cow milk, and cow milk samples contaminated with α-hemolysin (100 μg mL^–1^, 3 μM). As depicted in Figure 4D, a slight difference in the reporter output was registered between contaminated and noncontaminated milk, resulting in an intensity gain of 0.18; this difference was lower than expected according to previous experiments in buffer. We ascribed this effect to the high content of salts present in milk, leading to an increase of reporter flow through the strips and increasing the background signal. To improve these results, we draw inspiration from our previous findings on communication experiments in solution (Figure 3C) in which we determined that signal amplification increased when increasing the quantity of nanoparticles employed. Based on this, we prepared test strips as previously described but increasing the quantity of deposited nanoparticles from 5 to 50 μg. This increase in nanoparticle quantity resulted in an increase in the (background) control intensity. Yet, to our delight, the reporter output in contaminated milk samples significantly increased (Figure 4E), leading to a more noticeable discrimination between contaminated and noncontaminated milk, and resulting in a 3-fold increase in intensity gain (from 0.18 to 0.61) when increasing nanoparticle amount from 5 to 50 μg. Altogether, these results validate the communication system in complex biological samples, and provide a strategy to improve the performance of communication networks based on tuning the quantity of nanoparticles.

To summarize, we have presented here a new strategy for the design of sensing systems based on engineered chemical communication between micro/nanoparticles, in which senders emit a chemical messenger (when exposed to the target analyte) to induce the release of a reporter from distant receivers. We demonstrated this approach by synergistically combining cell-sized lipid vesicles and gated nanoparticles for sensing purposes. The use of engineered GUVs allows the release of a messenger upon analyte-membrane interactions, and gated nanoparticles translate this message into the release of a chromo-fluorogenic reporter. The communication system exhibited tunable signal amplification and allowed implementation on test-strips with smartphone detection. The applicability of our strategy was demonstrated using α-hemolysin as target analyte; yet, the experimental demonstration of other applications will be a matter of future work. Our work demonstrates that chemical communication between different types of particles can be applied in the development of sensing systems. Given the possibility to employ a wide variety of gating ensembles and to design tailor-made vesicle membranes composed of diverse building blocks (e.g., lipids, polymers, or protein conjugates), we believe this is a versatile approach that could be extended to other micro/nanoparticle platforms for sensing diverse analytes.

## Data Availability

Experimental details and additional data are available in Methods section and in the ESI. Any other data supporting this article are available from the corresponding authors upon request.

## Supporting Information Available

Materials, Instrumentation, Methods, and Figures S1 to S12 (PDF)
