# Supplementary material for: Chemical Communication between Giant Vesicles and Gated Nanoparticles for Strip-Based Sensing
Source: Nano Lett. 2024 Oct 23;24(44):14050–7. doi: 10.1021/acs.nanolett.4c04022 (PMC11544697; doi:10.1021/acs.nanolett.4c04022)
Supplement: Supplementary file 1 — nl4c04022_si_001.pdf [file nl4c04022_si_001.pdf]

Supporting information for

Chemical Communication between Giant Vesicles and Gated Nanoparticles for Strip-Based Sensing

Jordi Ventura-Cobos,[a] Estela Climent,[a,b,c] Ramón Martínez-Máñez,[a,b,c,d,e,] Antoni Llopis-Lorente [a,b,e,*]*

[a] Instituto Interuniversitario de Investigación de Reconocimiento Molecular y Desarrollo Tecnológico (IDM), Universitat Politècnica de València, Universitat de València, Camino de Vera s/n, 46022 València, Spain. *E-mail: rmaez@qim.upv.es, anllolo2@upvnet.upv.es.

[b] CIBER de Bioingeniería, Biomateriales y Nanomedicina (CIBER-BBN). Instituto de Salud Carlos III, 28029 Madrid, Spain.

[c] Unidad Mixta de Investigación en Nanomedicina y Sensores, Universitat Politècnica de València, Instituto de Investigación Sanitaria La Fe (IISLAFE), Avenida Fernando Abril Martorell, 106, 46026 Valencia, Spain.

[d] Unidad Mixta UPV-CIPF de Investigación en Mecanismos de Enfermedades y Nanomedicina, Universitat Politècnica de València, Centro de Investigación Príncipe Felipe, C/ Eduardo Primo Yúfera 3, 46100 Valencia, Spain.

[e] Departamento de Química, Universitat Politècnica de València, Camino de Vera s/n, 46022 Valencia, Spain.

1. Materials

1,2-dioleoyl-sn-glycero-3-phosphocholine (DOPC); 1-Palmitoyl-2-oleoyl-sn-glycero-3-phosphocholine (POPC), 1,2-distearoyl-sn-glycero-3-phosphoethanolamine-N-[methoxy(polyethylene glycol)-2000] (DSPE-PEG) and 1,2-dioleoyl-sn-glycero-3-phosphoethanolamine-N-(lissamine rhodamine B sulfonyl) (RhB-DOPE) were purchased from Avanti Polar Lipids. Tetraethyl orthosilicate (TEOS), n-cetyltrimethylammonium bromide (CTABr), sodium hydroxide (NaOH), tris(2,2′-bipyridyl) dichlororuthenium(II) hexahydrate $[Ru(bpy)_3]Cl_2 \cdot 6H2O$, (3-glycidyloxypropyl)trimethoxysilane, 3-aminophenyl boronic acid, cholesterol, paraffin oil (CAS Number: 8012-95-1), glucose, sucrose, bovine serum albumin (BSA), acetylcholinesterase from *Electrophorus electricus* (Type VI-S), acetylthiocholine iodide, 8-Hydroxypyrene-1,3,6-trisulfonic acid trisodium salt (HPTS) and α-hemolysin from *S. aureus* were purchased from Sigma Aldrich. Chambered microscope slides (μ-slide, 18 wells, glass bottom, 1.5H) were purchased from Ibidi. Grade GF/C Glass Microfiber Filter Papers were purchased from Whatman™.

2. Instrumentation

UV-visible measurements were performed using a JASCO V-760 spectrophotometer. Confocal laser scanning microscopy was performed using the Leica DMi8 confocal microscope. TEM images were acquired using a JEOLTEM-1010 Electron microscope working at 100 kV. Powder X-ray diffraction (PXRD) measurements were performed using a Seifert 3000TT diffractometer using CuK_{α} radiation. N_2 adsorption-desorption isotherms were recorded using a Micromeritics TriStar II Plus automated analyzer. Z-potential measurements studies were performed using a ZetaSizer Nano ZS (Malvern). Photographs of test strips were acquired using 3D-printed home-made case, equipped with a 465 LED as excitation source and a smartphone connected as showed in Figure S1. Pictures were recorded using a SAMSUNG Galaxy S6.

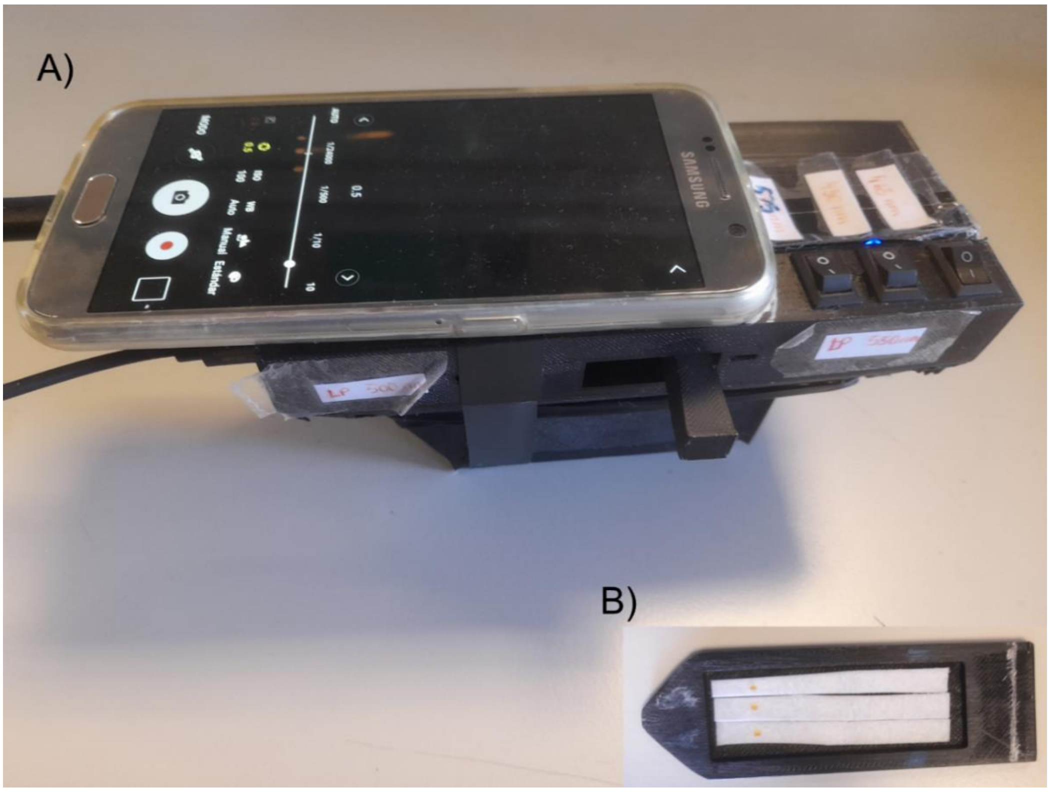

Figure S1. A) 3D-printed customized case, connected to a smartphone. B) Strip holder with strips, which is inserted into the 3D-printed case for image collection.

3. Methods for GUV preparation and characterization

Synthesis of giant unilamellar vesicles (GUVs)

DOPC, POPC and cholesterol stock solutions in chloroform were prepared at 100 mg mL^{-1}, DSPE-PEG was prepared at 10 mg mL^{-1} and RhB-DOPE was prepared at 1 mg mL^{-1}. All solutions were stored at -20 °C until use. To synthesize GUVs the droplet transfer method was used (Figure S2). First, DOPC, POPC and cholesterol, were mixed inside a glass vial (70 x 10 x 0.8-1.0 mm), to have a combined amount of 2 μmol. For the experiments related with the optimal proportion of cholesterol, the molar ratios of DOPC/POPC/Cholesterol used were 50/50/0, 35/35/30 and 25/25/50. For chemical communication, the molar ratio of DOPC/POPC/Cholesterol used was 25/25/50. DSPE-PEG in a 1% molar ratio was added to increase GUVs stability and prevent membrane fusion. On the other hand, RhB-DOPE was added when confocal imaging was needed in a 0.2% molar ratio. Next, 200 μL of paraffin oil were added to resuspend the lipids in a non-polar solution. In order to evaporate the chloroform content, the glass vials were incubated in a sand bath for 30 min at 80 °C, being vortexed 1-2 times for 5 seconds in between the incubation. Then, 20 μL of precooled inner phase (IP) solutions were introduced inside the 200 μL of paraffin-lipid solutions and vortexed for 30 seconds while tilting the glass tubes. For confocal imaging of GUVs, the IP was prepared using 0.2 M sucrose, PBS 1X (pH 7.5) and 50 μM HPTS. For chemical communication, the IP was prepared with 80 mM ATCh, 0.2 M sucrose and PBS 0.02X. Subsequently, glass tubes were incubated again on ice for at least 10 min to allow the lipid monolayer formation surrounding the water droplets. For confocal imaging of GUVs, the outer phase (OP, aqueous phase outside GUVs) was prepared using 0.2 M glucose and PBS 1X. For chemical communication, the OP was prepared using 0.2 M glucose, PBS 0.02X and 80 mM NaCl to prevent differences in the osmotic pressure. Afterwards, 150 μL of OP were

introduced in 1.5 mL Eppendorf tubes and precooled on ice. Subsequently, lipid emulsions were layered over the 150 µL OP containing Eppendorf tubes and incubated at 4 °C for 5-10 min to allow the interfacial lipid monolayer formation. Next, the tubes were centrifuged at 4 °C for 20 min at 3300 g. After centrifugation, the Eppendorf tubes were punctured with a 21-gauge needle at the position of the GUVs pellet and the aqueous phase was collected in a new Eppendorf tube. To remove any non-encapsulated compound, GUVs were washed 3 times by replacing the supernatant with 80 µL fresh OP and centrifugation at 2000 g for 2 min.

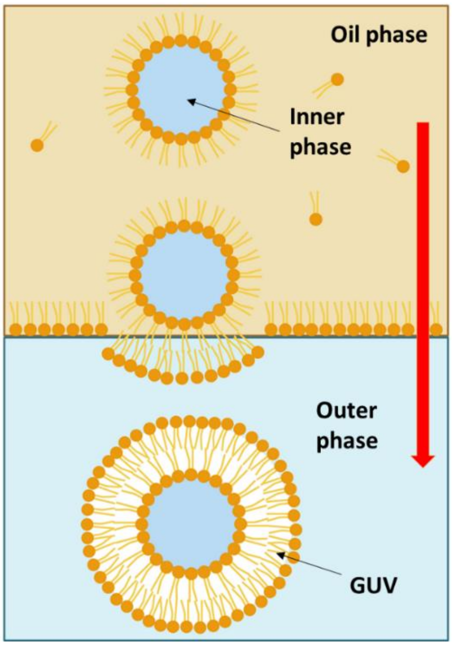

Figure S2. Schematic of the preparation of giant unilamellar vesicles by the droplet transfer method.

Cargo release from HPTS-loaded GUVs by confocal imaging

To visualize cargo release from GUVs by confocal imaging, a chambered microscope slide was used. First, the slide wells were passivated with 100 µL of 1 mg mL^{-1} bovine albumin serum (BSA) for 10 min and washed once with ultrapure water. Then, 50 µM

HPTS loaded GUVs were resuspended in 60 µl of OP. Next, 2.5 µL of the suspension of GUVs were introduced with 100 µL of OP in the wells. Afterwards, GUVs were let to settle at the bottom for 10 min. For imaging, the Leica TCS DMi8 confocal microscope was used. The encapsulated dye (HTPS) was excited at 488 nm, while the membrane marker (RhB-DOPE) was excited at 532 nm. Fluorescence emissions of HPTS and RhB-DOPE were collected at 500-530 and at 550-620 nm respectively. After the addition of α-hemolysin (20 µg mL^{-1}), GUVs were incubated for 10 and 60 min, and the percentage of permeabilized GUVs was studied (Figure S4). It was observed that 60 min increased the percentages of permeabilization for all the compositions. It is expected that eventually all the GUVs, regardless of their cholesterol ratio, would release their cargo, with the exception of multilamellar GUVs, or GUVs that are present inside other GUVs. Pictures were taken in the same plane with an ACS APO 40x/1.15 oil objective (512 x 512 resolution).

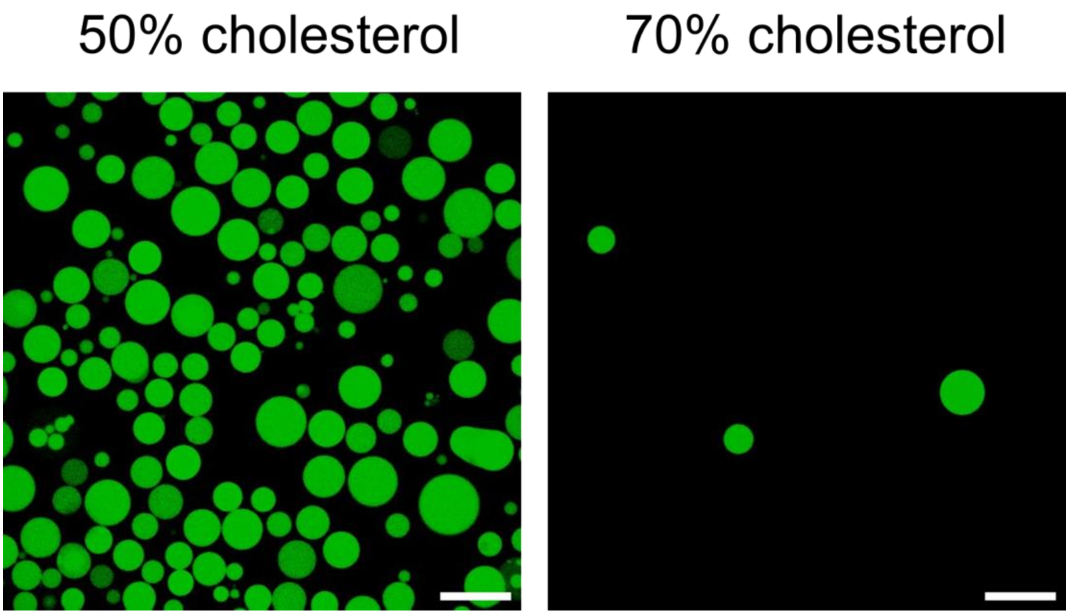

Figure S3. Confocal images of GUVs loaded with HPTS (50 µM) with 50% and 70% cholesterol proportion in their membranes. Scale bars represent 25 µm.

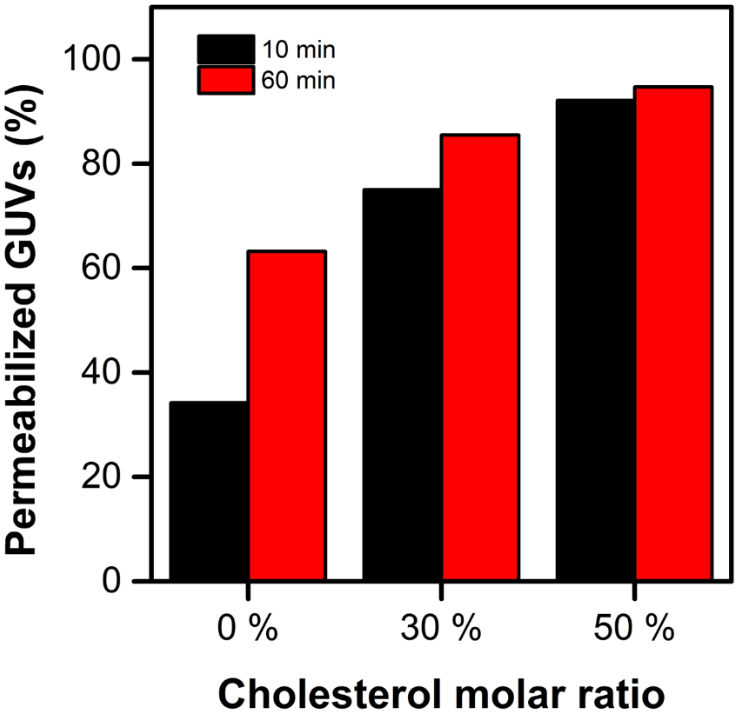

Figure S4. Percentage of permeabilized GUVs as a function of cholesterol molar ratio after 10 and 60 min of incubation with α-hemolysin (20 μg mL^{-1}, 0.6 μM), as determined by confocal microscopy analysis (N>250).

Quantification of the ATCh released from GUVs

Fist, the pellet of 80 mM ATCh-loaded GUVs was resuspended in 30 μL of OP as control, or with 30 μL of α-hemolysin (100 μg mL^{-1}, 3 μM) containing OP for 10 min. Later, samples were centrifuged at 2000 g for 2 min to precipitate the GUVs, and the 30 μL of supernatant were transferred to a new Eppendorf. To quantify the ATCh released, the Ellman´s reagent 5,5′-dithiobis-(2-nitrobenzoic acid (DNTB) was employed. To prepare the assay 100 μL of PBS 1X, 3 μL of 10 mM DTNB and 3 μL of AChE (0.1 mg mL^{-1}) were placed in a Eppendorf tube. A calibration curve was prepared by adding 10 μL of ATCh in different concentrations (from 1 μM to 100 μM). The reaction mixtures were incubated for 10 min under stirring to ensure the complete hydrolysis of ATCh into

thiocholine and acetic acid by AChE. DTNB is able to interact with the thiol group of thiocholine, which cleavages the disulfide bond of DTNB, generating a product (TNB^{2-}) with a maximum absorbance at 412 nm (Figure S5). After the 10 min incubation, the absorbance was measured at 412 nm. Following the same procedure, dilutions of the samples (1:10) of the ATCh released from GUVs were reacted with DTNB. The ATCh released was calculated by extrapolation with the calibration curve.

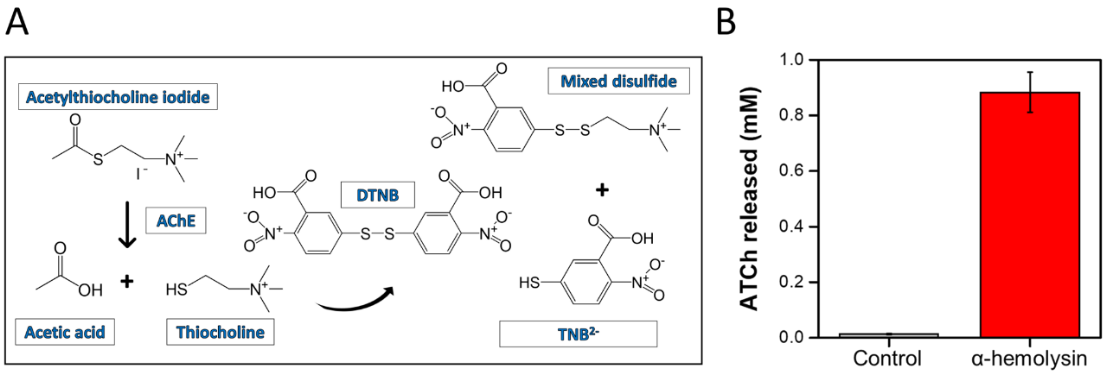

Figure S5. A) Chemical mechanism of the method developed for ATCh quantification, based on Ellman´s reaction. B) Quantified concentration of ATCh released from GUVs. Error bars correspond to the s.d. of three independent GUV preparations.

4. Methods for nanoparticle preparation and characterization

Preparation of gated mesoporous silica nanoparticles

First, 1 g (2.74 mmol) of n-cetyltrimethylammonium bromide (CTABr) was dissolved in 480 mL of deionized water and stirred for 20 min at 400 rpm. Then, the pH was basified by adding 3.5 mL of a 2 M NaOH solution and the temperature was increased to 80 °C. Once 80 °C were reached, 5 mL (22.4 mmol) of tetraethyl orthosilicate (TEOS) was added dropwise into the solution while maintaining the stirring. Magnetic stirring was kept for 2 h to give a white suspension. The solid was isolated and washed 3 times with water by centrifugation at 9500 rpm, until neutral pH was reached. Next, the solid was dried at 70 °C overnight. To obtain the final MCM-41-type mesoporous silica nanoparticles, the solid was calcined at 550 °C in an oxidant atmosphere for 5 h in order to remove the surfactant. To functionalize the silica surface, 100 mg of nanoparticles were resuspended in 15 mL of acetonitrile. Next, 500 µL of (3-glycidyloxypropyl) trimethoxysilane (2.26 mmol) were added and the mixture was stirred for 5.5 h at room temperature. Subsequently, the solid was isolated by centrifugation at 12000 rpm for 3 min and dried at 37 °C overnight. Then, the solid was resuspended in 15 mL of toluene and 154 mg (1.13 mmol) of 3-aminophenyl boronic acid were added. Afterwards, the suspension was left stirring overnight. Next, the solid was washed by centrifugation once with toluene and once with acetonitrile. To load the pores with the dye, 150 mg of $[Ru(bpy)_3]Cl_2$ were added to the suspension of nanoparticles in 15 mL of acetonitrile and stirred overnight. Then, the solid was washed with toluene twice and dried at 37 °C overnight. To do the capping with the enzyme acetylcholinesterase, 0.5 mg of enzyme and 5 mg of nanoparticles were suspended in 500 µL of 50 mM phosphate buffer at pH 7.5. Acetylcholinesterase is a glycoprotein, thus it binds to nanoparticles by the formation of cyclic phenylboronic acid esters between the oligosaccharide chains of the protein and the phenylboronic groups.

The mixture was stirred overnight at 4 °C to prevent enzyme degradation. Finally, the resulting nanoparticles were washed twice, resuspended in 500 µL of phosphate buffer and stored at -20 °C until use. The molecular gate (gatekeeper) formation and its chemical structure is represented in Figure S6. The acetylcholinesterase-gated nanoparticles can recognise and hydrolyse acetylthiocholine into thiocholine and acetic acid, decreasing the pH and inducing cargo release from the nanoparticles.

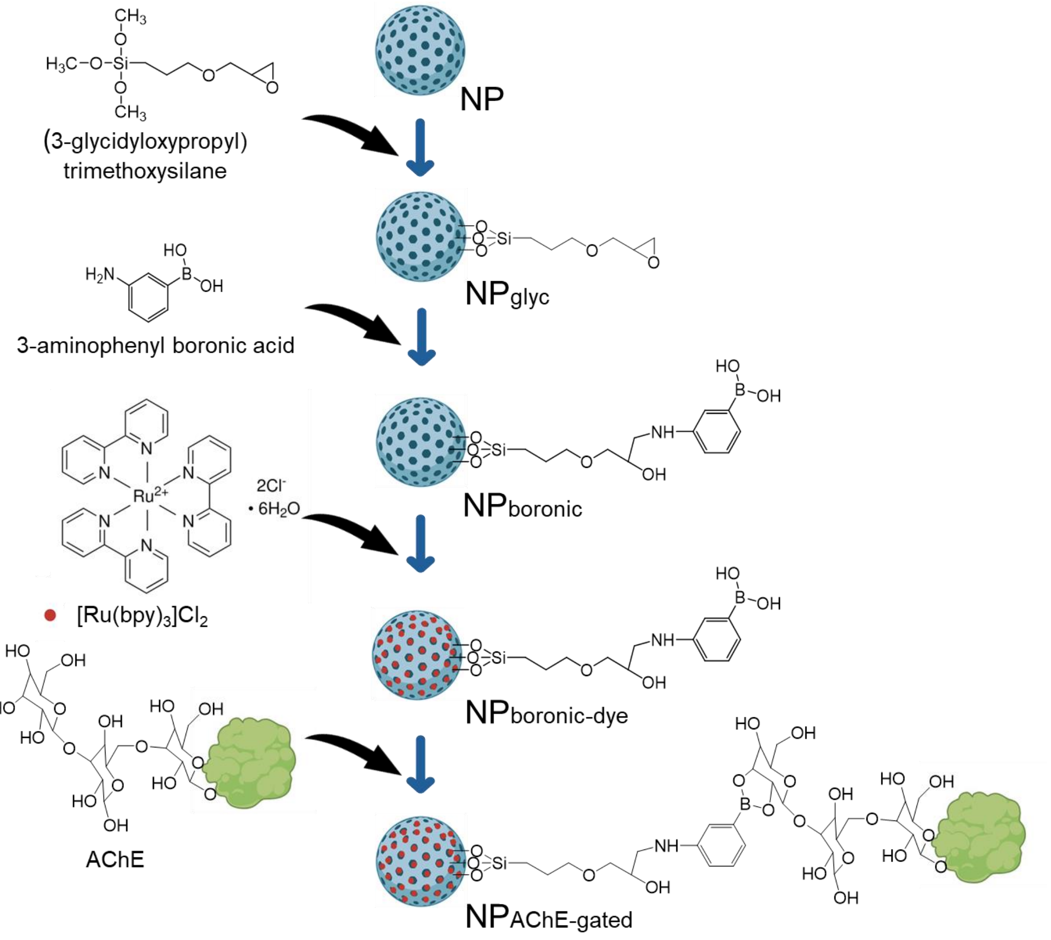

Figure S6. Schematic representation of the different functionalization steps and corresponding chemical structures for the construction of AChE-gated nanoparticles.

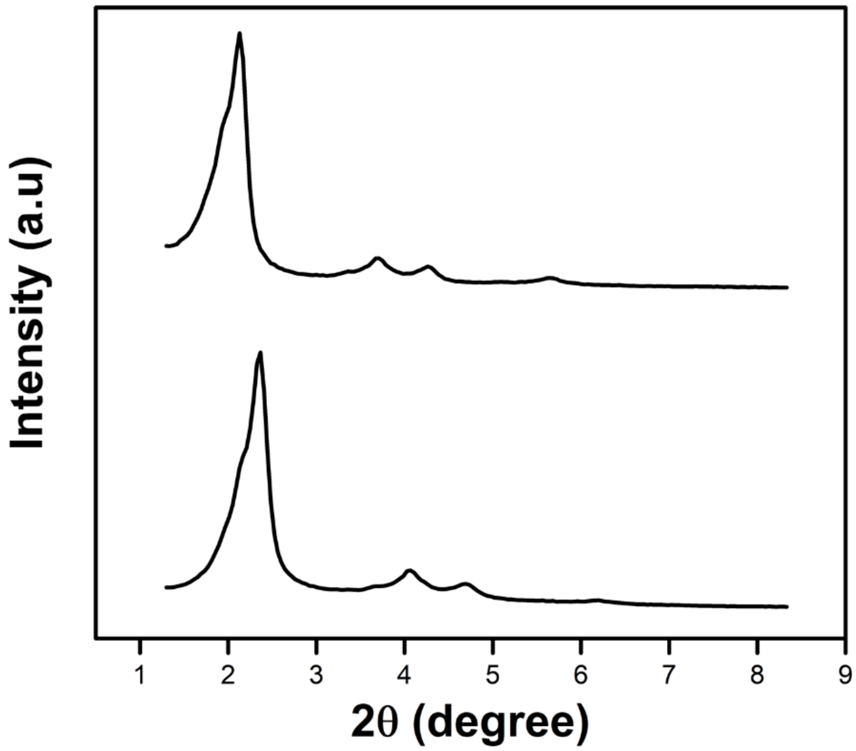

Figure S7. Powder X-ray diffraction patterns of mesoporous silica nanoparticles before (top pattern) and after (bottom pattern) the removal of the surfactant by calcination at 550 °C. The nanoparticles before calcination present the characteristic low-angle reflection peaks of MCM-41 type mesoporous scaffolds. The peaks after calcination were slightly displaced to the right, which relates to the condensation of silanol groups after calcination.

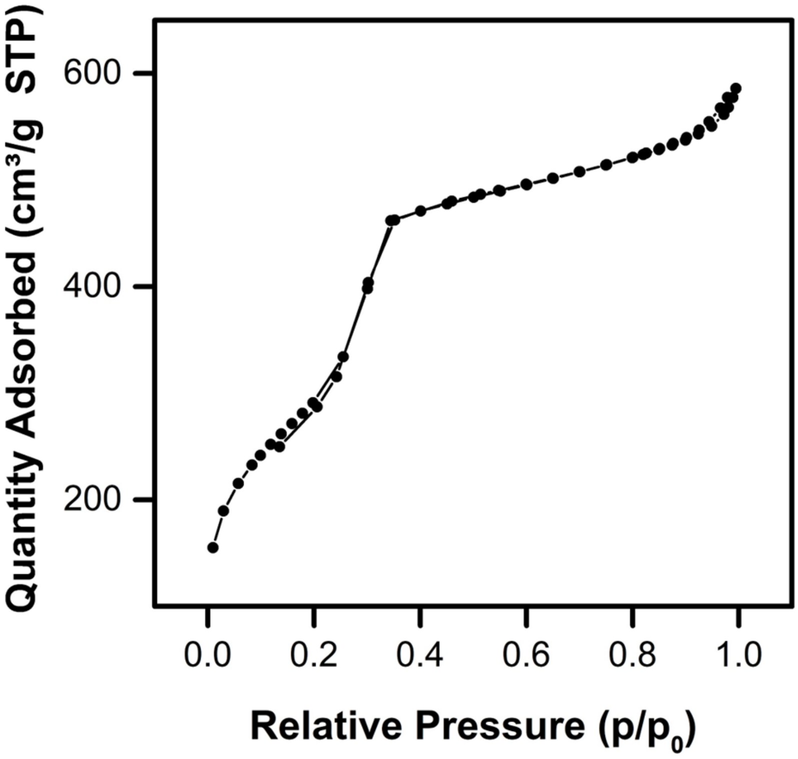

Figure S8. N_2 adsorption-desorption isotherms for the calcined nanoparticles.

The N_2 adsorption-desorption isotherm, represented in figure S7, showed the characteristic adsorption step at P/P_0 of 0.3 for mesoporous silica nanoparticles with empty pores, related with the nitrogen condensation inside the pores by capillarity. The total specific surface calculated using the BET model was 1118 m^2 g^{-1}. On the other hand, the means of the pore volume and size, obtained using the BJH model associated to adsorption inside the pores, were 1.01 cm^3 g^{-1} and 3.08 nm respectively.

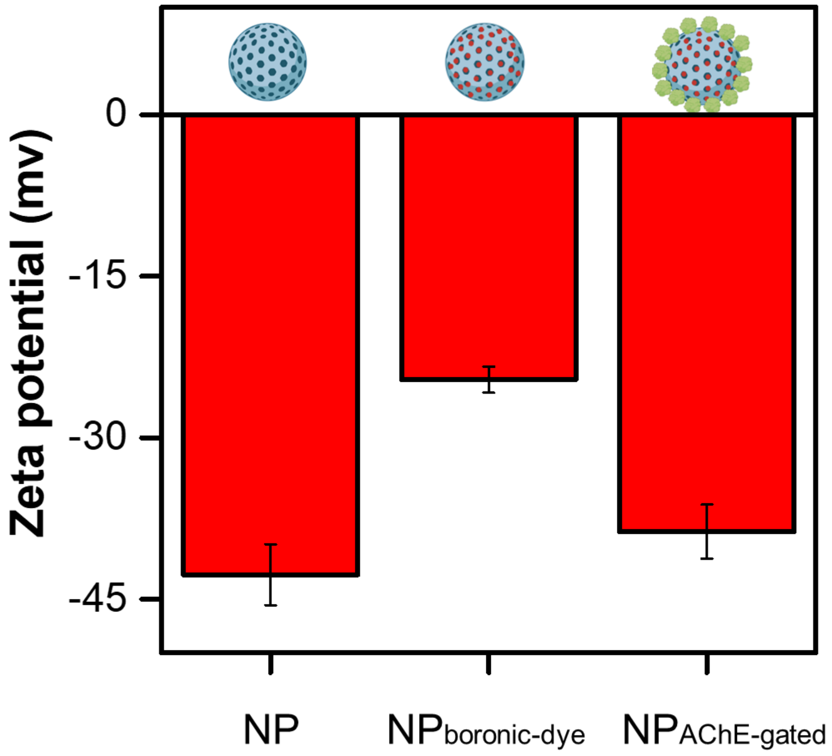

Figure S9. Zeta potential of mesoporous silica nanoparticles (NP), functionalized and dye loaded-nanoparticles ($NP_{boronic\text{-}dye}$), and the final device with the AChE anchored to the surface ($NP_{AChE\text{-}gated}$). Error bars correspond to the s.d. of three independent measurements.

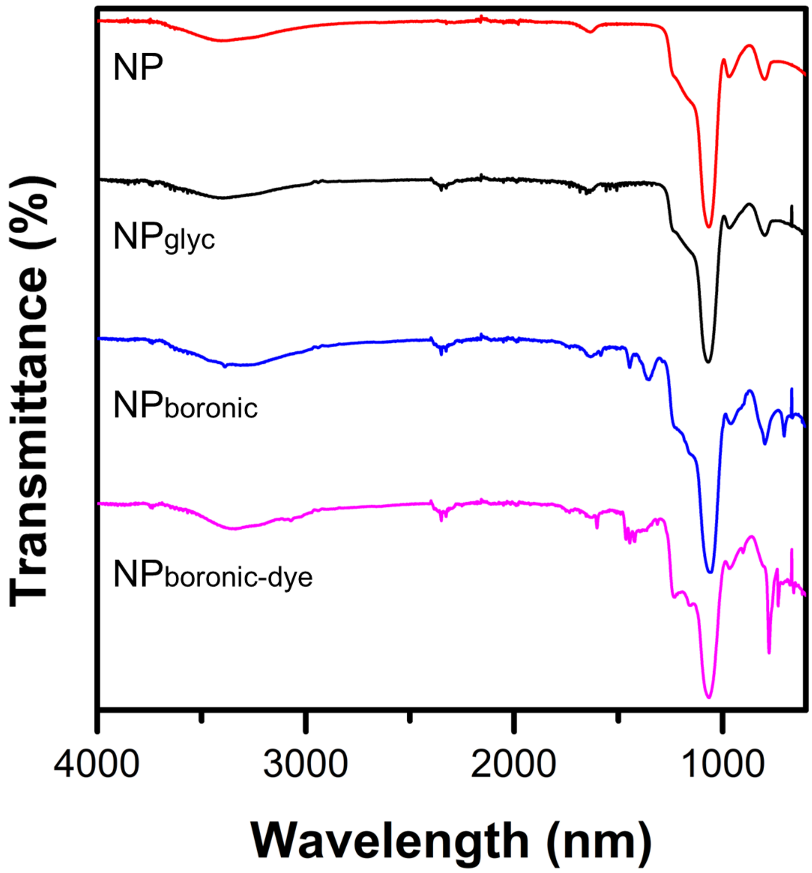

Figure S10. FTIR spectra of mesoporous silica nanoparticles (NP, red), after (3-glycidyloxypropyl) trimethoxysilane treatment (NP_{glyc}, black), after 3-aminophenylboronic acid treatment ($NP_{boronic}$, blue) and after $[Ru(bpy)_3]Cl_2$ loading of nanoparticles ($NP_{boronic\text{-}dye}$, pink).

The FTIR spectra of the mesoporous silica nanoparticles, represented in figure S9, showed a major band at ca. 1080 cm^{-1}, corresponding to the Si-O stretching vibrations. After treatment with 3-aminophenylboronic acid, new peaks appear at ca. 1500-1400 and 700-600 cm^{-1}, which can be ascribed to the vibration of C-C and C-H aromatic bonds, and B-O bonds. Finally, after $[Ru(bpy)_3]Cl_2$) loading, the spectrum showed new peaks corresponding to bipyridine rings at ca. 1600-1400, 1250-1150 and 700 cm^{-1}.

Quantification of the total amount of encapsulated dye

The total amount of encapsulated dye was estimated by stirring the nanoparticles (0.1 mg) for 1 h in pH 2.5, acidified with HCl. Samples were stirred at 900 rpm in a thermoshaker. Then, samples were centrifuged at 12000 rpm for 3 min to isolate the solid and absorbance in the supernatant was measured at 452 nm. The total amount of encapsulated dye was estimated to be 76.5 µg mg^{-1}.

The amount of $[Ru(bpy)_3]Cl_2$ released was calculated by applying the formula:

$$\left(C = \frac{A}{\varepsilon}\right)$$

Where, C is the concentration (M), A is the absorbance at 452 nm, and ε is the molar extinction coefficient of $[Ru(bpy)_3]Cl_2$ at 452 nm (14.6 mM^{-1} cm^{-1}).

Enzymatic activity of the nanoparticles

The immobilization of the enzyme on the surface of gated nanoparticles was confirmed by running an enzymatic assay. The formation of TNB^{2-} as a function of time was monitored for 3 min using a UV-visible spectrophotometer. To prepare the assay 100 µL of PBS 1X, 3 µL of 10 mM DTNB and 2 µL of 0.2 M ATCh were placed in a quartz cuvette. Then, either 15 µL of PBS 1X (control) or gated nanoparticles (0.1 mg mL^{-1}) were added and mixed by pipetting. Finally, the absorbance was measured at 412 nm for 3 min. The enzymatic units were calculated using the following formula:

$$\frac{Enzymatic\ Units}{g} = \frac{(\Delta - \Delta_{blank}) * V_T}{(\varepsilon_{TNB} * \mathrm{l} * \mathrm{V}_{NPs} * \mathrm{C}_{NPs})}$$

Where, Δ is the slope of the graph (min^{-1}), Δ_{blank} is the slope of the graph for the blank (min^{-1}), V_T is the total volume in the cuvette, ε_{TNB} is the molar extinction coefficient of

TNB^{2-} at 412 nm (13.7 mM^{-1} cm^{-1}), l is the optical path in the cuvette (1 cm), V_{NPs} is the volume of nanoparticles added (mL), and C_{NPs} is the concentration of nanoparticles suspension added (g mL^{-1}).

ATCh-responsive reporter release from gated nanoparticles

Stock solutions of gated nanoparticles (10 mg mL^{-1}) were washed 3 times with PBS 0.02 X at pH 7.5. Later, gated nanoparticles were resuspended at a concentration of 2 mg mL^{-1} in media consisting of 100 µL PBS 0.02 X supplemented (or not) with 20 mM ATCh. The samples were stirred at 25 °C and 900 rpm in a thermoshaker, and absorbance measurements at 452 nm were taken at scheduled times. Previous to each measurement, samples were centrifuged at 12000 rpm for 3 min. The amount of $[Ru(bpy)_3]Cl_2$ released was calculated by applying Beer-Lambert law. Release percentage was calculated taking into account the total amount of encapsulated dye (previously determined as described above). As shown in Figure S11, absorbance and fluorescence intensity of $[Ru(bpy)_3]Cl_2$ does not change between pH 7.5 (starting pH in solution) and pH 5 (final pH after ATCh hydrolysis).

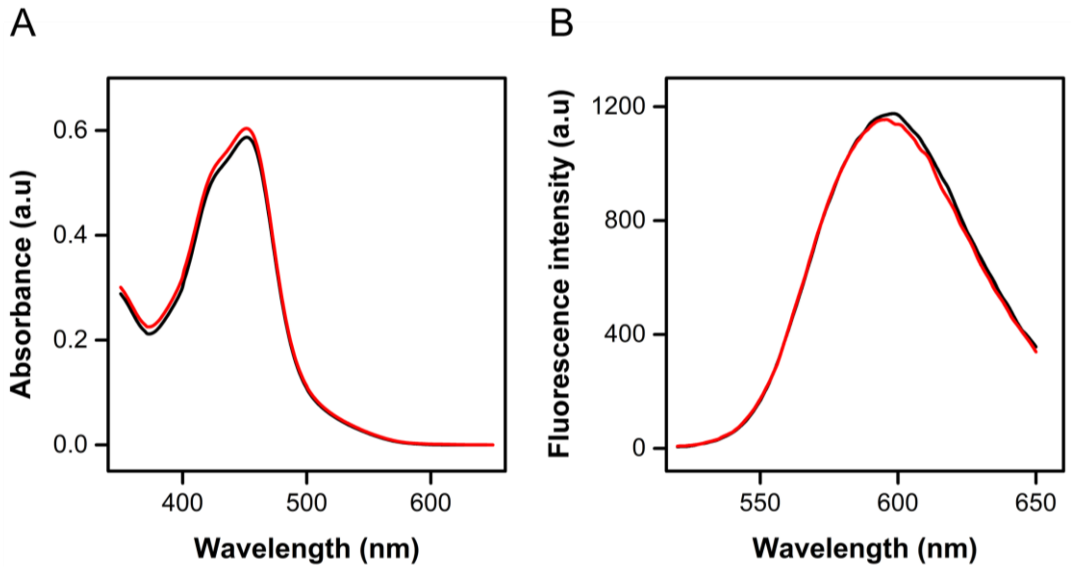

Figure S11. (A) Absorbance and (B) fluorescence intensity (λ_{exc}=453 nm) spectra of 40 μM $[Ru(bpy)_3]Cl_2$ at pH 7.5 (black line) and at pH 5 (red line).

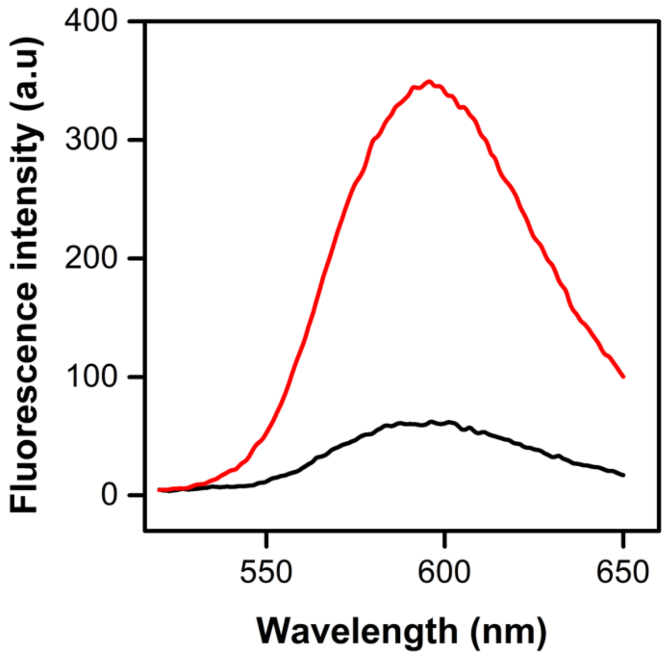

Figure S12. Fluorescence intensity (λ_{exc}=453 nm) spectra of $[Ru(bpy)_3]Cl_2$ released from gated nanoparticles in aqueous solution after 10 min incubation at pH 7.5 (black) and at pH 5 (red).

5. Methods for chemical communication experiments in aqueous media

First, the pellet of 80 mM ATCh-loaded GUVs was resuspended in 30 µL of OP as control, or with 30 µL of α-hemolysin (100 µg ml^{-1}, 3 µM) containing OP for 10 min. Later, samples were centrifuged at 2000 g for 2 min to precipitate the GUVs, and the 30 µL of supernatant were transferred to solutions containing the gated nanoparticles to study their cargo release. Stock solutions of gated nanoparticles (10 mg mL^{-1}) were washed 3 times with PBS 0.02 X at pH 7.5. Later, gated nanoparticles were resuspended at a concentration of 2 mg mL^{-1} in the reaction mixtures consisting of 100 µL PBS 0.02 X and the 30 µL of GUV supernatant. The samples were stirred at 25 °C and 900 rpm in a thermoshaker, and absorbance measurements at 452 nm were taken at scheduled times. Previous to each measurement, samples were centrifuged at 12000 rpm for 3 min. The amount of $[Ru(bpy)_3]Cl_2$ released was calculated by applying the formula:

$$\left(C = \frac{A}{\varepsilon}\right)$$

Where, C is the concentration (M), A is the absorbance at 452 nm, and ε is the molar extinction coefficient of $[Ru(bpy)_3]Cl_2$ at 452 nm (14.6 mM^{-1} cm^{-1}). Release percentage was calculated taking into account the total amount of encapsulated dye (previously determined as described above).

6. Methods for strip-based lateral flow experiments

In a first step to prepare the strips, two double-side tape film (2.5 cm wide) were attached to a plastic sheet, one below the other, to have a covered surface with tape film of 5 cm wide. Next, the covers were removed, and a Glass Microfiber membrane (Glass Fiber GF/C grade; Whatman ® Paper sheet) was attached over the tape film. Afterwards, 5 µL of gated nanoparticles suspension (1 mg mL^{-1}) were deposited at 1 cm of the border, with a distance of 0.5 cm between each droplet. After drying the spots of nanoparticles for 5 min, individual strips were cut to have a size of 0.5 x 5 cm. If needed, strips could be stored at 4 °C for up to 4 weeks.

For a strip-based test, the strips were introduced and incubated for 5 min in Eppendorfs containing increasing concentrations of ATCh for characterization experiments, or the pellet of GUVs with a total volume of 120 µL for communication experiments. When studying the cargo release at increasing concentrations of ATCh, the samples consisted of PBS 0.02 X at pH 7.5 and ATCh at different concentrations. When studying the cargo release at increasing concentrations of α-hemolysin, the samples consisted of the outer phase of GUVs with different concentrations of α-hemolysin. When studying the cargo release in milk, the samples consisted of the 90 µL of outer phase and 30 µL of milk. After 5 min elution, the strips were introduced in a 3D-printed customized holder (Figure S1). The fluorescence of the dye released was measured with a smartphone, while using a LED emitting at 465 nm, a short-pass filter at 500 nm to remove the residual light source from LED and a long-pass filter at 550 nm to efficiently collect the fluorescent emission of reporter dye released along the membrane. The collected images from strips were analyzed using the program ImageJ, by analyzing the integrated fluorescence density of

the red channel from samples, while correcting the background fluorescence of strips. The limit of detection was calculated using the following formula:

$$LOD = K * \frac{Sb}{m}$$

Where, K = 3, Sb is the standard deviation of the blank, and m is the slope of the curve.
